# The immunology of parasite infections: Grand challenges

**DOI:** 10.3389/fpara.2022.1069205

**Published:** 2022-11-08

**Authors:** Sheila Donnelly

**Affiliations:** School of Life Sciences, University of Technology Sydney, Sydney, NSW, Australia

**Keywords:** parasite, immunology, immune-modulation, microbiota, helminth-therapy, vaccine

## Introduction

Parasitic diseases continue to be a major cause of morbidity and mortality worldwide, with billions of people at risk of infection, often with multiple parasites (https://www.who.int). Despite this global impact, there are limited treatment options for most infections and only one human vaccine, which is moderately effective against malaria (Stutzer et al., 2018; Zavala, 2022). The key to controlling parasite infection is to better understand the host immune response to these pathogens and to determine how this can be exploited to protect the host from infection and/or the pathological consequences of infection. However, this is a formidable task. By virtue of a dependency on a host for cyclical transmission and millennia of co-evolution with their hosts, parasites have developed multifaceted, often life-cycle stage-specific strategies to utilize, evade, modulate, and/or regulate the host immune response to avoid elimination and ensure their long-term survival to mature and complete their life cycle (Yazdanbakhsh and Sacks, 2010).

Through decades of research many of these mechanisms have been characterized (Chulanetra and Chaicumpa, 2021), and can be subdivided into different tactical approaches including, an ability to become camouflaged by host factors, inhabiting immune privileged sites, expressing stage specific antigens, secreting components that mimic host factors, driving the differentiation of immune cell phenotypes, and regulating the responsiveness of host immune cells. Importantly, these studies have provided critical insights into fundamental mechanisms of human immunology. Most notably, murine experimental leishmaniasis was the first model to confirm the biological significance of the Th1/Th2 paradigm *in vivo* (Scott, 1989). While these discoveries have significantly contributed to our current knowledge of the interaction between parasites and their host’s immune response, there is still an incomplete understanding of this relationship. Future research will be informed by concomitant developments in immunological, molecular and cell culturing techniques and innovations, such as immune-metabolomics, cell signaling systems, imaging mass spectrometry, organoid culture, microfluidics, vaccine technology (and others). However, there are also important considerations that are specific to parasite infection, and I believe represent some of the grand challenges facing this field of parasitology.

## Grand challenge 1: Are parasite infections a necessary evil – is there a future for parasite derived therapeutics?

While parasites have clearly benefited from the development of immune-modulatory mechanisms, this evolutionary adaptation to support their long-term survival may have unwittingly positioned them as a necessary component of the human immune response (Fumagalli et al., 2009; Jackson et al., 2009). Due to their ubiquitous nature in human populations, it has been proposed that their presence provided the regulatory networks to keep activation of immune responses in check and drive the homeostatic resolution of inflammation. As a result of this co-dependence, the removal of parasites from human populations through increased sanitation and urbanization establishes an imbalance in the immune system, thereby increasing susceptibility to the inappropriate activation of inflammatory responses, resulting in immune-mediated disease (Jackson et al., 2009; Rook, 2010; Allen and Maizels, 2011). Indeed, there is compelling epidemiological evidence of an inverse correlation between the prevalence of endemic helminth infections and the incidence of immune-mediated disease in human populations worldwide. Furthermore, there is now a substantial body of experimental evidence in animal models showing that live parasite infection, or the administration of parasite-derived proteins/peptides, can prevent progression of inflammatory disease (Maizels, 2020; Ryan et al., 2020). Understandably, much of this research has been performed from the perspective of parasitology research, rather than a translational, clinical pathway. However, this means that the most appropriate pre-clinical models of immune-mediated disease have not always been utilized and that fundamental knowledge of the pharmacokinetics, mechanisms of action, and toxicity of the parasite molecules is often lacking (Sobotková et al., 2019).

The challenge here will be the establishment of a concerted, collaborative effort between research and industry partnerships. This will more effectively inform research activity to better position parasite-derived therapeutics as a consideration for clinical applications by the pharmaceutical industry and drive this solid (and encouraging) evidence of efficacy from bench to bedside.

## Grand challenge 2: Parasites do not live alone – a holistic approach is needed

In the same way the modulation of immune responses by parasites may impact the course of immune-mediated diseases, parasites can also significantly alter the developing immune response to concurrent infections, which consequently impacts the progression and severity of illness, and vice versa (Hassan and Blanchard, 2022). This is a particular concern for endemic human populations (and most animals), in which parasite infections occur against a background of co-infection with other parasites and other microbial pathogens (Venter et al., 2022). However, there is no clear consensus as to which concurrent infections are protective or which increase the severity of disease, nor is there a clear understanding on how the presence of multiple pathogens specifically influences the host immune response (Mabbott, 2018). Despite the important consequences of co-infection, the potential interactions among parasites are rarely considered in either clinical settings or in immunological analysis of models of infection. However, understanding how the host immune response behaves in this setting is critical to informing possible strategies for treatment and/or infection control.

In addition to the presence of pathogenic organisms, trillions of commensal microorganisms, inhabit the mammalian body. This microbiota underpins the health of its host with hypothesized impacts on inflammation, immune development, and disease outcomes. Evidence is emerging that the host microbiome plays a contributory role in the parasite’s modulation of host immune responses. Multiple studies have now shown that the presence of parasites in the intestine changes the overall composition of bacterial species in the gut (Zaiss and Harris, 2016; Chabé et al., 2017), which in turn influences inflammation and health (Ramanan et al., 2016; Coakley and Harris, 2019). Whether the host immune response to the parasite infection is altering the resident microbiota, and/or whether the parasites and their secreted products are directly modifying the composition of the microbiome remains to be elucidated.

In addition to the host microbiota, parasite-associated microbiota also shapes the host immune response and the pathological outcome to disease (Ives et al., 2011; Yurchenko and Lukeš, 2018). However, despite the identification of the *Wolbachia* endosymbiont of filarial nematodes more than 20 years ago (Williams et al., 2000), only a handful of parasite species have had their associated microbiome characterized (Dheilly et al., 2019). As a result, there is limited insight into the functional relationship between the parasite, its microbiome and the host and the consequences that this has upon the immune response and the outcome to infection.

This evidence of the involvement of microorganisms in the parasite-host relationship is a reminder of the complexity of a mammalian biological community. Disentangling the interactions between parasites and hosts, their respective microbiota, and microbial pathogens in the parasite’s direct environment, and understanding how these influence the host immune response will be a challenge. A holistic approach is required, accounting for all contributors, to elucidate the critical determinants of risk and benefit to host health.

## Grand challenge 3: The importance of the host – is the lab mouse an adequate model?

The majority of research elucidating the fundamental mechanisms of parasite immune modulation/evasion, has been performed in experimental animals, particularly rodents. This methodology has been driven by the broad availability of different strains (and transgenics) of mice and a multitude of murine specific reagents to support the characterization of immunological networks and cellular phenotypes. Although this approach has been central to the identification and characterization of immune responses during infection, it has likely only illuminated a small fraction of the heterogeneity in host-parasite immune relationships.

While laboratory mice have served well as models of mammalian immune function, generating many successful translational therapies to the clinic, it has been now well established that the immune cell populations in these mice are not equivalent to adult mammals and thus do not always exhibit corresponding immune responses to infection (Abolins et al., 2017; Masopust et al., 2017). The phenotype and functional activity of an animal’s immune response to infections is informed by their environment. In the laboratory setting, mice are generally housed and maintained in filtered, microbial free, controlled environments which is a contributing factor to the immunological divergence between lab mice and wild mice (and other mammals) (Graham, 2021), a difference which has been shown to influence the outcome of parasite infection. While the lab strain of C57BL/6 mice are resistant to high infectious doses of the intestinal nematode, *Trichuris muris*, when these mice were exposed to outdoor environments, they displayed a reduced capacity to produce Th2 type immune responses and as a result their susceptibility to infection was significantly increased (Leung et al., 2018). The factors that contributed to this change in immune response were not determined, but it is a scenario that should be extended to the study of other parasite infections.

Despite the vast number of identified helminths and protozoan parasites (Cox, 2002), our assumptions regarding the immune response to parasite infection has largely been formed from studies of only a handful of parasites that have the capacity to infect a laboratory mouse. Furthermore, the primary mechanisms of immune modulation attributed to helminths has generally been elucidated through infections with nematodes that have only adapted to infect a rodent host (*Heligmosomoides polygyrus*, *Trichuris muris).* While these may have been proven to be an adequate model for human intestinal helminth infection, there is no equivalent murine model for many of the larger tissue dwelling helminth parasites from which to elucidate immune modulatory mechanisms throughout their life cycle, as the tissue damage that ensues during maturation and migration of the adult parasites in these cases is often too severe for a murine host. For these parasites, large animal hosts are required to fully understand the host-parasite immune relationship, but the logistics, ethics, cost, and lack of suitable analytical reagents is often prohibitive.

To establish an infection in these models (mouse and large animal), in general, a single large infectious dose is administered to the host and the immune response and corresponding pathogenesis is tracked over time. Once again, although this is a necessary approach to gain some insight into the developing immune response to the presence (and development) of a pathogen, it is unlikely to represent a naturally occurring infectious dose. As such, it is important to consider how the pathology resulting from a high parasite burden is impacting the developing immune response and determine whether this is producing an artificial representation of the true parasite-host relationship. In addition, this experimental protocol of infection may be negatively impacting the evaluation of vaccine candidates. The potent immune modulation induced by such high infectious doses of parasites likely suppresses the antigen-specific responses induced by vaccination. In contrast, a low dose (or trickle dose in field trials for animal hosts) would better represent the real-world challenge for a vaccine.

Combined, these observations highlight the challenge in selecting models of parasite infection that authentically reflect that of their natural hosts and therefore accommodate the characterization of the true phenotype and functional activity of the host immune response during all stage of infection.

## Grand challenge 4: Expanding the characterization of parasite-host communication

A critical aspect to uncovering the mechanisms by which parasites control their host’s immune response is to characterize the communication signals used by the parasite. Accordingly, over several decades the excretory/secretory (ES) products of many parasites have been characterized. Starting with the identification of the proteins released by the parasites, the development of modern techniques enabled more in-depth characterization and expanded the repertoire of secreted molecules to glycans, lipids, miRNAs, small molecules and metabolites (Maizels et al., 2018; Ryan et al., 2022). Many of these components are homologous to host molecules involved in the regulation of immunity, thus endowing them with the capacity to mimic host signaling networks to modulate the immune response (Smyth et al., 2018, Ricafrente et al., 2021). Furthermore, some of these parasite derived molecules are secreted by the parasite within extracellular vesicles (EV), which are taken up by host cells (Wu et al., 2019), thus creating a cross-species channel of communication and facilitating the parasites with access to the intracellular machinery of host immune cells. A greater understanding of the biogenesis of parasite EVs and the selection and sorting of their cargo will transform our understanding of the parasite-host relationship, build on the existing knowledge of mechanisms of parasite immune modulation, and provide new targets for anti-helminthic strategies.

## Grand challenge 5: The characterization of a protective immune response - development of an effective vaccine

Ultimately, the study of parasite immunology is driven by the ambition of identifying effector mechanisms to eliminate the parasite and prevent future infections. However, despite the extensive characterization of the parasite’s mechanisms of immune modulation and immune evasion, the ability and capacity to therapeutically intervene in these infections remains rudimentary. Fundamentally, development of an effective parasite vaccine remains hampered by a lack of understanding of what constitutes a protective immune response. Although multiple clinical trials have suggested efficacy of putative vaccine candidates, most have not translated to the field.

Perhaps it is time to challenge thinking and the approach to parasite vaccine development. One question that needs to be asked is how good a vaccine against any specific parasite needs to be? After all, the only human parasite vaccine on the market is estimated to provide just a short-lived protection of ~34% (Zavala, 2022), an efficacy that would not be accepted by any standard vaccine development program. However, by reducing severe disease in young children, and when administered in conjunction with other control measures it may decrease exposure to the parasite, and therefore the vaccine will contribute to minimize or eliminate the clinical consequences of infection. So rather than trying to achieve resistance, perhaps the goal should be reimagined with the aim of minimizing or eliminating the pathological consequences of infection.

## Invitation to the immunology and immune evasion section of frontiers in parasitology

These grand challenges are not an exhaustive list, and as such The Frontiers in Parasitology Immunology and Immune Evasion Section is calling on the research community to initiate further conversation, to inspire and to provide informed guidance regarding the advances and research questions that are most important to them. Accordingly, we have released a call for reviews covering any topic in Parasite Immunology and Immune Evasion and currently have two open Research Topics named “Insights into Recent Advances and Future Perspectives in Parasite Immunology” and “Helminth Infection and Regulation of Unrelated Diseases”. We look forward publishing experimental work, clinical trials, perspectives, opinions, and reviews in this field of research.

## Author contributions

The author confirms being the sole contributor of this work and has approved it for publication.

## Conflict of interest

The author declares that the research was conducted in the absence of any commercial or financial relationships that could be construed as a potential conflict of interest.

## Publisher’s note

All claims expressed in this article are solely those of the authors and do not necessarily represent those of their affiliated organizations, or those of the publisher, the editors and the reviewers. Any product that may be evaluated in this article, or claim that may be made by its manufacturer, is not guaranteed or endorsed by the publisher.
